# Prenatal transmission of severe acute respiratory syndrome coronavirus 2, resulting in neonatal severe acute pneumonia, from an asymptomatic mother: a case report

**DOI:** 10.1186/s13256-023-04120-8

**Published:** 2023-09-07

**Authors:** Narcisse Elenga, Maman Rabiou Aboubacar Ahidan, Régine Buende Eyenga, Dandjeffo Houadjeto

**Affiliations:** 1 Pediatric Medicine and Surgery, Andrée Rosemon Regional Hospital, Cayenne Hospital, Rue des Flamboyants, BP 6006, 97306 Cayenne Cedex, French Guiana; 2 Department of Pediatrics and Neonatology, Saint Laurent-du-Maronil Hospital, Avenue Paul Castaing, 97320 Saint-Laurent du Maroni, French Guiana; 3 Department of Radiology, Saint Laurent-du-Maronil Hospital, Avenue Paul Castaing, 97320 Saint-Laurent du Maroni, French Guiana

**Keywords:** COVID-19, Prenatal transmission, Neonate, Severe acute pneumonia, Asymptomatic mother

## Abstract

**Background:**

We report a case of prenatal coronavirus disease 2019, which evolved 6 days after birth into severe pneumonia with signs of multiple organ failure, in a mother with asymptomatic coronavirus disease 2019.

**Case presentation:**

At minute 11 of life, our patient from Afro-Caribbean had polypnea with mild signs of struggle; Silverman’s index was scored at three. Chest radiography showed bilateral opacities consistent with respiratory distress syndrome. On the 6th day of life, a thoracic computed tomography scan showed bilateral parenchymatous lesions (10–20%) in ground glass, compatible with coronavirus disease 2019-type infection. At the same time, the neonate showed signs of multiple organ failure (elevated liver and cardiac enzyme levels). She was treated with azithromycin (20 mg/kg/day) for 5 days. All the signs recovered fully by day 12. Real-time polymerase chain reaction results were positive in the first 30 min of life, suggesting prenatal transmission. Our patient has been followed until 2 years old and is developing well with no sequelae.

**Conclusion:**

This case report demonstrates the incompatibility between maternal asymptomatic coronavirus disease 2019 and severe neonatal lung involvement. We emphasize the need for vigilance to avoid missing the most severe forms of neonatal coronavirus disease 2019.

## Background

Given the low incidence [1] of antepartum or per partum transmission of severe acute respiratory syndrome coronavirus 2 (SARS-CoV-2), there is a need to describe such cases to better understand them. Although neonatal infection with SARS-COV2 is usually not severe, vigilance is required to avoid missing the more serious forms, which may be life-threatening [2, 3]. Here, we report a case of prenatal coronavirus disease 2019 (COVID-19), which evolved 6 days after birth into severe pneumonia with signs of multiple organ failure, in a mother with asymptomatic COVID-19.

## Case presentation

### Maternal

In June 2021, a 22-year-old woman (gravida 1, para 1, no history of diabetes, hypertension, sickle cell disease, or any other disease, no surgical history) was admitted to our institution at 38 weeks gestation for labor. The patient was not vaccinated against SARS COVID-19. The prenatal course was uncomplicated. Ultrasound surveillance results at 25, 28, and 32 weeks were normal. She tested negative for toxoplasmosis, other infections (syphilis, varicella-zoster, parvovirus B19), rubella, cytomegalovirus (CMV), herpes (TORCH), human immunodeficiency virus (HIV), and hepatitis B. She tested positive for group B streptococcus vaginal infection and was treated according to French guidelines. She was afebrile and asymptomatic with normal vital signs throughout pregnancy. The duration of water break was less than 12 h. Four days before delivery, she routinely tested positive for SARS-CoV-2, without any symptoms.

### Neonatal outcome

**Fig. 1:**
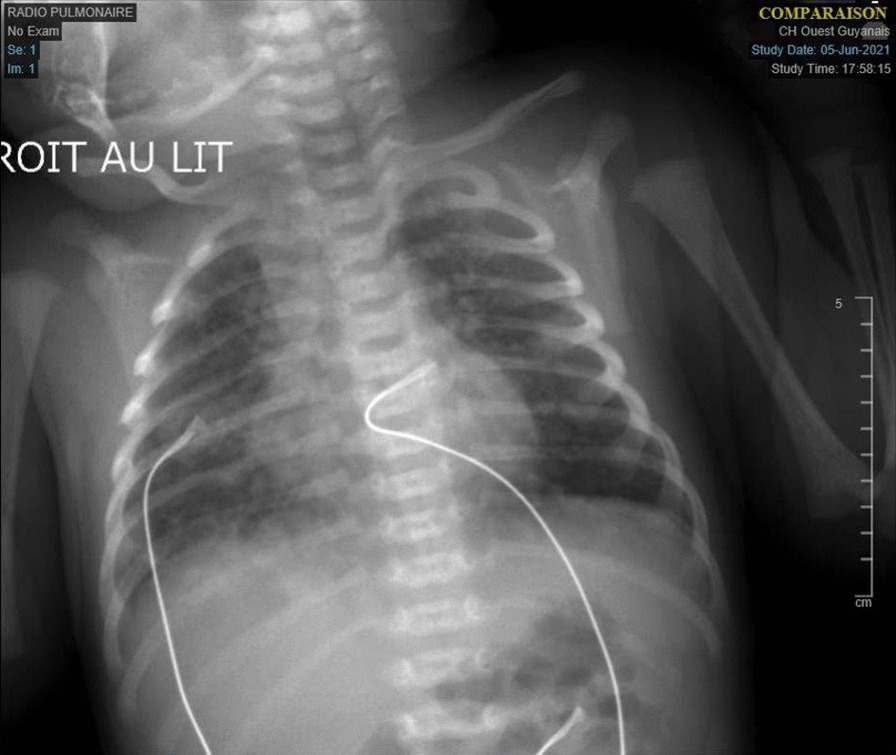
Chest radiography showed bilateral opacities

**Fig. 2:**
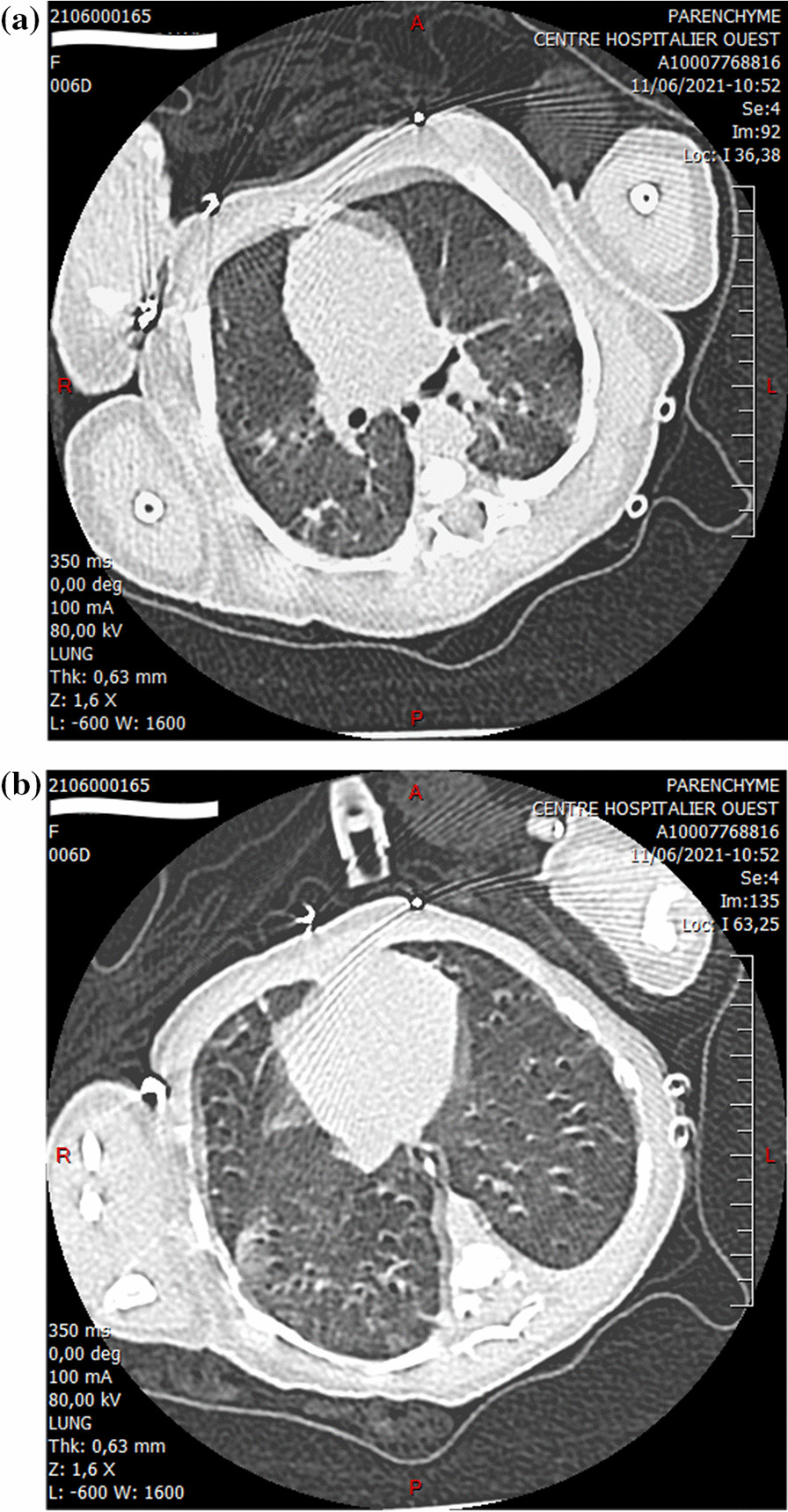
**a** Computed tomography scan showing bilateral parenchymatous lesions (10–20%), in ground glass, compatible with a coronavirus disease-type infection. **b** This figure also shows ground glass pictures

A live-born female infant was delivered vaginally. The resuscitation and newborn APGARs were 10 and 10 at 1 and 5 min, respectively. Birth weight was 2670 g (18th percentile), length was 49 cm (63rd percentile), and head circumference was 32 cm (10th percentile), all appropriate for gestational age. The patient’s physical status at birth was unremarkable. At minute 11 of life, she presented with respiratory distress (tachypnea, nasal flaring, chest retractions, grunting, and saturation of 90%) requiring noninvasive ventilation. The Silverman index was 3. Peripheral pulses were well perceived, and there was no heart murmur. The rest of the physical examination results were unremarkable. Arterial blood gas (ABG) showed pH 7.30, PCO_2_ of 60 mmHg, PaO_2_ of 65 mmHg, and HCO_3_^–^ of 20 mmol/L. Thirty minutes after delivery, the neonatal nasopharyngeal swab was positive for SARS-CoV-2 real-time polymerase chain reaction (RT-PCR), and immunoglobulin (Ig)-M and IgG for SARS-CoV-2 were negative. Neonatal isolation was implemented immediately after birth without mother–neonate skin-to-skin contact. At minute 12 of life, she was transferred to the neonatal intensive care unit (ICU) to continue noninvasive ventilation and explore for respiratory distress. At 30 min, chest radiography revealed bilateral opacities consistent with respiratory distress syndrome (Fig. 1). Intravenous penicillin plus gentamycin antibiotics were started and stopped after 5 days, as blood cultures, cerebrospinal fluid remained negative, and C-reactive protein (CRP) levels were low. On the 6th day of life, faced with the persistence of the respiratory distress, a thoracic computed tomography (CT) scan was performed, showing bilateral parenchymatous lesions (10–20%) in ground glass, compatible with a COVID-19-type infection (Fig. 2). At the same time, the neonate showed signs of multiple organ failure (elevated liver and cardiac enzyme levels). The liver enzymes alanine transaminase (ALT), aspartate transaminase (AST), and lactate dehydrogenase (LDH) were 60 IU/L, 540 U/L, and pro-BNP > 350 ng/L, respectively (Table 1). No signs of coronary artery dilation were observed on echocardiography. She was treated with azithromycin (20 mg/kg/day) for 5 days. All the signs recovered fully by day 12. Results of repeated neonatal PCR sampling were positive on days 1, 3, 10, and 18. Inflammatory tests (CRP, Pro-BNP, and LDH), as well as blood gas performed at 2 weeks of age, were strictly normal. She was discharged 30 days after birth, with a plan for close follow-up by her pediatrician. The COVID-19 RT-PCR test result was negative on day 30. At the 3-month follow-up, the infant was breastfeeding well with no fever or respiratory distress. Six months later, mother and baby were doing well. Our patient has been followed for 6 months and has normal growth. She is now 2 years old and developing well, with no sequelae.

**Table 1:** Results of laboratory workup

Characteristic	Patient’s values	Normal values
Hemoglobin	14.8 g/dL	12.5–20.5 g/dL
MCV	102 fL	86–124 Fl
MCH	35.3 pg	28–40 pg
MCHC	34.7/dL	28–88
Leukocytes	7.92 G/L	5.0–21.0 G/L
Neutrophils	2.8 G/L	1.0–9.5 G/L
Lymphocytes	3.2 G/L	2.0–17.0 G/L
Monocytes	1.8 G/L	0.1–1.7 G/L
Eosinophils	0,14 G/L	0.0–0.85 G/L
Basophils	0.01 G/L	0.0–0.10 G/L
Platelets	632 G/L	170–500 G/L
Erythrocytes	4.19 T/L	3,6–6,2 T/L
TP	93%	70–100%
TCA	34.3 s	25.1–36.5 s
INR	1.05	
Serum albumin	28 g/L	38–54 g/L
Fibrinogen	g/L	2.38–4.98 g/L
K^+^	5.7 mmol/L	3.50–5.10
Na^+^	137 mmol/L	136.0–145.0
Cl^–^	101 mmol/L	98.0–107.0
Urea	0.7 mmol/L	2.8–8.1 mmol/L
Creatinine	27 μmol/L	39–60 μmol/L
Glycemia	4.0 mmol/L	5.85–6.05 mmol/L
AST	60 U/L	5–34 UI/L
ALT	18 U/L	0–55 UI/L
GGT	86 U/L	9–36 UI/L
ALP	121 U/L	< 500 UI/L
LDH	540 U/L	125–220 UI/L
CRP	12 mg/L	< 5 mg/L
PCT	0.08 ng/Ml	< 0.5 ng/Ml
Ferritin	μg/L	15.0–80.0 μg/L
D-dimer	–	(0.00–0.05)
Pro-BNP	2417 ng/L	< 125 ng/L
Troponin	70.7 ng/L	< 15.6 ng/L
TSH	3.96 mUI/L	0.35–4.94 mUI/L
T4	23.1 pmol/L	9.1–19.5 pmol/L
T3	8.4 pmol/L	2.65–9.68 pmol/L
Cerebrospinal fluid	Normal	

*MCV* Mean Corpuscular Volume, *MCH* Mean Corpuscular Hemoglobin, *MCHC* Mean corpuscular hemoglobin concentration, *PT* Prothrombin time, *APTT* activated partial thromboplastin time, *INR* International Normalized Ratio, *AST* aspartate transaminase, *ALT* alanine transaminase, *GGT* glutamyltransferase, *ALP* alkaline phosphatase, *LDH* lactate dehydrogenase, *CRP* C-reactive protein, *PCT* procalcitonnine, *Pro-BNP* prohormone of brain natriuretic peptide, *TSH* thyroid-stimulating hormone

## Discussion and conclusion

This clinical case confirms severe neonatal COVID-19 infection with prenatal transmission [4]. Although this is a rare situation, vertical transmission of COVID-19 is no longer questionable. Of concern is the delay in the diagnosis of severe COVID-19 in neonates whose mothers are not diagnosed before delivery. In our case, the mother was not diagnosed until a few days before delivery, although she had no symptoms of COVID-19. Indeed, testing for maternal COVID-19 should be systematic and repeated during pregnancy even in asymptomatic women. Knowing that this mother was COVID-19 positive allowed us to perform a SARS-CoV2 RT-PCR on the newborn and to know that the latter was also infected with COVID-19. Although the differential diagnosis of this neonatal respiratory distress initially led us to think of it as transient respiratory distress, it was moderate in a neonate who did not have fetal distress, as evidenced by the Apgar score up to 10 min of life. The meconium aspiration syndrome (MAS) could also be evoked, as it was found to be higher in the COVID-19 cohort [5]. However, the initial clinical presentation was not favorable. Finally, the persistence of respiratory distress and the presence of biological signs of inflammation completed by the thoracic CT scan enabled us to confirm the diagnosis of COVID-19 pneumonia. It is rare, but it is important to know that the intensity of pneumonia and the level of pulmonary involvement in newborns can be inconsistent with the severity of the illness in mothers infected with COVID-19 [3, 6].

Transplacental transmission of SARS-CoV-2 has been well documented [7]. However, it has been shown that the human receptor ACE2, which transports the virus into host cells, is widely expressed in the placenta. Its expression increases with the trimester of pregnancy and it can transfer the virus transplacentally to the fetus during the last trimester of gestation [8, 9].

The scientific community, especially pediatricians, must be vigilant regarding these rare forms of severe COVID-19 in newborns to avoid fatal outcomes [3]. These neonatal infections acquired late in pregnancy do not usually result in intrauterine growth retardation or prematurity, especially in healthy mothers [10]. An infection acquired a few days before birth, which sometimes develops in utero, can become complicated a few days later, resulting in severe symptoms after birth. Given the rarity of these cases, one question remains unanswered: What are the factors favoring the severity of COVID-19 in these newborns?

In conclusion, our case report demonstrates the incompatibility between asymptomatic maternal COVID-19 and severe neonatal lung involvement. Therefore, we emphasize the need for vigilance to avoid missing the most severe neonatal COVID-19 cases.

## Data Availability

Data are available on reasonable request.

## Abbreviations

SARS -CoV-2: Severe acute respiratory syndrome coronavirus 2
COVID‐19: Coronavirus disease 2019
TORCH: Toxoplasmosis, other (syphilis, varicella-zoster, parvovirus B19), rubella, cytomegalovirus (CMV), herpes
HIV: Human immunodeficiency virus
PCR: Polymerase chain reaction
RT-PCR: Real-time reverse-transcription PCR
ICU: Intensive care unit
CRP: C-reactive protein
CT: Computed tomography
MAS: Meconium aspiration syndrome
